# Abnormal late postprandial glucagon response in type 1 diabetes is a function of differences in stimulated C-peptide concentrations

**DOI:** 10.3389/fendo.2024.1419329

**Published:** 2024-08-01

**Authors:** Lingyu Zhang, Yao Qin, Yiting Huang, Qizhen Hu, Qian Wu, Xing Wang, Mei Zhang

**Affiliations:** ^1^ Department of Endocrinology, The First Affiliated Hospital with Nanjing Medical University, Nanjing Medical University, Nanjing, Jiangsu, China; ^2^ Department of Endocrinology, The Affiliated Changzhou Second People’s Hospital of Nanjing Medical University, Nanjing Medical University, Changzhou, Jiangsu, China

**Keywords:** type 1 diabetes, alpha cell regulation, glucagon, stimulated C-peptide, late glucagon response

## Abstract

**Background:**

The functional changes in alpha cells in patients with type 1 diabetes (T1D) with different residual beta cell functions remain poorly elucidated. The study aimed to investigate the relationship between glucagon secretion and C-peptide levels and to explore the relationship between glucagon response and glucose increment in respond to a secretagogue in a steamed bread meal tolerance test (BMTT) in T1D.

**Methods:**

The study enrolled 43 adult patients with T1D and 24 healthy control subjects. Patients with T1D who underwent BMTT were divided into two groups based on peak C-peptide levels: C peptide low (CPL; C-peptide < 200 pmol/L; n=14) and high (CPH; C peptide ≥ 200 pmol/L; n=29). Plasma glucose, C-peptide, glucagon levels at 0, 30, 60, 120, and 180 min were measured. The glucagon response to the BMTT was defined by areas under the curve (AUC) as early (AUC_0-30_), late (AUC_30-180_), or total (AUC_0-180_) glucagon.

**Results:**

Compared to healthy individuals, fasting plasma glucagon was lower and postprandial plasma glucagon level was increased in patients with T1D. Glucagon levels after BMTT between the CPL and CPH group showed significant group by time interaction. Peak glucagon and glucagon at 60-180 min, total and late glucagon response were higher in CPL than CPH group, while fasting glucagon and early glucagon response adjusted for glucose were comparable between CPL and CPH group. The higher late glucagon response and late glucagon response adjusted for glucose were associated with lower peak C-peptide in T1D. The higher late glucagon response and lower peak C-peptide were associated with the higher value of ▵glucose at 180 min.

**Conclusion:**

Stimulated C-peptide levels affect the paradoxical increase in postprandial glucagon secretion in patients with T1D, especially late glucagon response. The exaggerated postprandial glucagon secretion further stimulates the elevation of postprandial glucose in patients with T1D.

## 1 Introduction

Blood glucose homeostasis is mainly regulated by pancreatic islet hormones, primarily insulin and glucagon. Insulin secretion by pancreatic beta cells has been intensively studied for its impact on glucagon secretion by pancreatic alpha cells under physiological conditions. The insulin receptor and its downstream signaling proteins are abundantly expressed in alpha cells, allowing insulin to suppress glucagon secretion (1). However, in pathological conditions, this negative feedback balance is disrupted due to impaired beta cell function. Patients with diabetes have been shown to exhibit insufficient suppression of glucagon secretion following oral ingestion of glucose intake or a meal (2, 3). Consequently, abnormalities in glucagon physiology may contribute to the development of fasting and postprandial hyperglycemia in the pathogenesis of type 1 diabetes (T1D) and its therapy.

Many studies have investigated the beta cell heterogeneity in T1D (4). However, clinical and immunologic characteristics of T1D vary significantly between different populations (5). For example, Chinese adults with newly diagnosed T1D have been reported to display high C-peptide levels (6). Our recent study further confirmed that Chinese patients with T1D exhibited substantial residuals beta cell mass despite ongoing autoimmune attacks (7). Despite this, the understanding of the stimulus-secretion coupling of alpha cell function and the residual beta cell function in response to an oral glucose challenge in T1D remains limited, with conflicting data emerging (8, 9). While previous studies had reported a significant exacerbation of postprandial hyperglucagonemia during the first one and five years after T1D diagnosis (9–11), two studies suggested that residual dysregulated glucagon secretion is not affected by beta cell function in T1D (12, 13). Thus, the primary question that arises is whether stimulated C-peptide levels could result in differential glucagon responses.

The phase of glucagon secretion in diabetes after oral ingestion of glucose intake or a meal remains unclear, with only a few studies focusing on type 2 diabetes (T2D). Early glucagon response, rather than late glucagon response, at baseline in non-diabetic individuals was significant associated with increased fasting glucose levels over 7 years (14). Additionally, the loss of early glucagon response suppression after oral glucose intake is only observed in T2D patients compared to healthy and pre-diabetes individuals, supporting the hypothesis that hyperglycemia in T2D is mainly related to impairment of the early glucagon response (15). These findings suggest that the levels of glucagon secretion following glucose load is crucial for the maintenance of normoglycemia. However, there are currently no studies that have looked in depth at the glucagon response in the early and late postprandial glucagon response in patients with T1D. Therefore, the second question of concern is whether the phases of postprandial glucagon secretion is associated with stimulated C-peptide and glucose increment in patients with T1D.

The aim of this study is to improve our understanding of the relationship between different phases of glucagon secretion and C-peptide concentrations and to investigate the relationship between the phases of glucagon response and glucose increments in response to a steamed bread meal tolerance test (BMTT) in Chinese patients with T1D.

## 2 Materials and methods

### 2.1 Study population

The present observational study was carried out in the Department of Endocrinology of the First Affiliated Hospital of Nanjing Medical University, Nanjing, China. Two types of subjects were enrolled in the study: 43 patients with T1D and 24 healthy control subjects. Major eligibility criteria for the patients with T1D included 1) age ≥ 18 years at the time of screening, 2) clinical diagnosis of T1D, 3) positivity for at least one islet antibody (IAA, ICA, GADA, or IA-2A) (16), and 4) able to provide written informed content. Exclusion criteria included 1) diabetic ketoacidosis or severe hypoglycemia within study preceding one month; 2) severe chronic diabetic complications (including proliferative retinopathy, autonomic neuropathy, macrovascular or central nervous system disease); 3) pregnancy or lactation, and 4) history of gastrointestinal surgery or pancreatectomy. The healthy controls were recruited from hospital and university staff, and the inclusion criteria for the healthy controls included 1) age≥ 18 years at the time of screening; 2) normal glucose tolerance, and 3) no family history of diabetes.

Patients with T1D were divided into two groups according to the peak serum C-peptide level after BMTT. The BMTT has been used most often in China as a measurement tool to evaluate beta cell function during follow-up after individuals have been diagnosed with diabetes (6, 17). Patients with T1D with a peak serum C-peptide level below 200 pmol/L were defined as the C-peptide low (CPL) group. Patients with T1D and peak serum C-peptide values above 200 pmol/L were divided into a C-peptide high (CPH) group.

The study was approved by the local ethics committee of the First Affiliated Hospital of Nanjing Medical University (approval no. 2019-SR-121.A1). The study was carried out in accord with the principles expressed in the Declaration of Helsinki.

### 2.2 Experimental procedures

The BMTT was performed after an overnight fast, with no food or drink other than water from midnight. Prior to the study, the patients achieved satisfactory glycemic control for three consecutive days. Fasting glucose was measured using a glucometer before the initiation of the BMTT, ensuring that the glucose level was targeted within the range of 4-10 mmol/L (72-180 mg/dL). If the fasting glucose level is not within the target range, the BMTT will be rescheduled. Patients with multiple daily insulin injections (MDIs) were instructed to take usual long-acting insulin dose the night before the study, while patients with continuous subcutaneous insulin infusion (CSII) maintained their basal insulin infusion. The delivery of continuous subcutaneous insulin was halted 2 hours prior to the initiation of the BMTT. Participants were instructed not to administer premeal bolus insulin and any correction dose of rapid-acting insulin during the BMTT. The BMTT, provided by the First Affiliated Hospital of Nanjing Medical University, containing 75 g of glucose, approximately 7 g of protein, and 1 g of fat, amounting to a total of 337 kcal. Blood samples were obtained at 0, 30, 60, 120, and 180 min during the BMTT. Throughout the entire procedure, and continuous monitoring of glucose levels was conducted. After the 180-minute BMTT, the determination of the necessary dosage of rapid-acting insulin required to maintain glucose levels within the desired target range was promptly performed by a physician until the target range was achieved.

### 2.3 Laboratory methods

Serum glucose was measured with an automatic enzymatic analyzer (Beckman Coulter, USA). Serum C-peptide levels were measured by a chemiluminescence assay (Roche Diagnostics, Switzerland) with a detection limit of 3·33 pmol/L. Islet autoantibodies IAA, IA-2A, and GADA were measured by ELISA (Euroimmun Medizinische Labordiagnostika AG, Germany; Biomerica, USA). An indirect immunofluorescence technique was used to measure ICA autoantibodies (18).

EDTA tubes containing aprotinin (0.6 TIU/ml of blood) were used to collect blood samples for plasma glucagon measurements. Blood samples were centrifuged for 15 min immediately after collection and stored at -80°C. Plasma glucagon was analyzed with a solid phase two-site enzyme immunoassay (Mercodia, Sweden), which has a detection limit of 1 pmol/L. The coefficient of variation (CV) for intra-assay variation was 3·3–5·1%, and the CV for inter-assay variation was 7·3–9·4% (19).

### 2.4 Calculations

The change in glucagon levels at 30, 60, 120, and 180 min during the BMTT (▵glucagon 30, 60, 120, and 180 min) was determined by comparing the glucagon levels at these time points to the baseline (0 min) glucagon level, as previously described (20). The change in glucose levels during the BMTT was calculated using the same method as ▵glucose 30, 60, 120, and 180 min. The glucagon response following the BMTT was expressed as the incremental area under the curve (iAUC), which were calculated using GraphPad Prism software (version 7.0). The iAUC from 0-180 and 0-30 min were calculated using the fasting value as the baseline, while the iAUC from 30-180 min was calculated using the 30min value as the baseline. Areas above the baseline were recorded as positive and areas below the baseline as negative. The iAUC from 0-180, 0-30 and 30-180 min was defined as the total, early and late glucagon response, respectively. In order to evaluate the glucagon response adjusted for glucose increment during the BMTT, we also calculated the ratio of iAUC glucagon to iAUC glucose from 0-180, 0-30 and 30-180min as previously described (21).

### 2.5 Statistical analysis

All statistical analysis were performed using IBM SPSS Statistics 22 and GraphPad Prism (version 7.0) software. Statistical significance for the parameter estimate was established with an alpha of 0.05.

#### 2.5.1 Analytical approach

The normal distribution of continuous variables was assessed using the Shapiro-Wilk test or Kolmogorov-Smirnov test. Unpaired t test or Mann-Whitney U test were used to compare the difference in clinical characteristics, iAUC of glucagon, the ratio of iAUC glucagon to iAUC glucose, and fasting variables between two groups, where appropriate. The differences between the two groups in the repeated measured variables following the BMTT were compared using the generalized estimation equation (GEE) approach to indicate the effect of time, group, and group by time interaction with baseline measurement (0 min) as covariates. An exchangeable working correlation matrix was applied in the GEE approach to assess change over time. The group-by-time interaction, which indicates the difference for given variables between two groups following the BMTT, was tested first. If significant, between-group differences at each timepoint were tested.

Relationships between variables were evaluated by spearman’s rank correlations. Multiple linear regression analyses with backward elimination were performed to determine the association of glucagon response and peak C-peptide. Model 1 was applied with iAUC _30-180_ glucagon as the dependent variable and including the following independent variables: peak C-peptide, sex, age, BMI, HbA1c, duration of diabetes, daily insulin dose, glucose 0, and peak glucose. Model 2 was applied with iAUC _30-180_ glucagon/iAUC _30-180_ glucose as the dependent variable and including the peak C-peptide, sex, age, BMI, HbA1c, duration of diabetes, and daily insulin dose as independent variables. Multiple linear regression analysis was also used to further explore the relationship between different phases of glucagon response and ▵glucose 180 min, with sex, age at diagnosis, and daily insulin dose as the covariates.

#### 2.5.2 Sensitivity analyses

To assess the robustness of glucagon levels analyses during the BMTT, two analytical approaches involving multiple covariates were performed (**Table 1**; **Supplementary Table 2**). The first approach included baseline measurement (0 min) as covariates in the GEE model; The second approach included clinical characteristics that differed between T1D and HC groups (age) or CPL and CPH groups (age and duration of diabetes) as additional covariates in the GEE model.

**Table 1:** Sensitivity analyses of the glucagon levels during the BMTT in participants with type 1 diabetes divided by peak C-peptide levels.

	Glucagon, pmol/L		Group×time interaction effect	CPL group vs. CPH group	
	CPL group (n=14)	CPH group (n=29)		Adjusted mean difference (95% CI)	*P* value
Multiple imputation					
0 min	4·20 ± 2·24	4·47 ± 2·28	0·003		
30 min	11·05 ± 5·66	8·32 ± 4·03		2·93 (-0·01 to 5·87)	0·051
60 min	11·07 ± 6·31	6·72 ± 3·41		4·55 (1·53 to 7·57)	0·003
120 min	10·31 ± 6·31	4·72 ± 2·37		5·79 (2·68 to 8·90)	<0·001
180 min	8·95 ± 5·03	4·31 ± 2·09		4·85 (2·41 to 7·29)	<0·001
Multiple imputation with adjustment for age, duration of diabetes and baseline measurement					
0 min	4·20 ± 2·24	4·47 ± 2·28	0·003		
30 min	11·05 ± 5·66	8·32 ± 4·03		2·11 (-0·61 to 4·84)	0·128
60 min	11·07 ± 6·31	6·72 ± 3·41		3·73 (1·12 to 6·34)	0·005
120 min	10·31 ± 6·31	4·72 ± 2·37		4·97 (2·26 to 7·68)	<0·001
180 min	8·95 ± 5·03	4·31 ± 2·09		4·03 (1·86 to 6·21)	<0·001

In the sensitivity analyses, the robustness of the results was assessed using 2 different analytical approaches. The repeated measured glucagon levels following the BMTT between two groups were investigated by generalized estimating equations. The first approach included baseline measurement (0 min) as covariates; The second approach included age, duration of diabetes and baseline measurement (0 min) as covariates. A significant group×time interaction indicated a significant difference for glucagon levels between two groups during the BMTT in all 2 approaches. BMTT, steamed bread meal tolerance test; CPL, C-peptide low; CPH, C-peptide high.

## 3 Results

### 3.1 Elevated glucagon response after the BMTT in T1D

A total of 43 patients with T1D (24 male and 19 female) and 24 healthy control subjects (10 male and 14 female) were enrolled in the study (**Supplementary Figure 1**). The sex and BMI of participant did not differ substantively between the T1D and healthy control groups, while patients with T1D showed higher median age compared with healthy control group (**Table 2**).

**Table 2:** Key clinical characteristics of type 1 diabetes and healthy control.

Characteristic	T1D group	HC group	*P* value
Subjects, n	43	24	NA
Male/Female, n	24/19	10/14	0·267^a^
Age, years	31·23 (23·81; 46·25)	23·95 (23·40; 27·75)	0·020
Duration of diabetes, years	3·00 (0·58; 7·00)	NA	NA
BMI, kg/m^2^	20·33 ± 2·59	21·68 ± 3·41	0·073

Results were expressed as mean ± SD or median (25th; 75th). Variables were compared using the unpaired t test or Mann-Whitney U test; ^a^Table was analyzed using Chi-squared test. NA, not applicable; T1D, type 1 diabetes; HC, healthy control.

**Figure 1:**
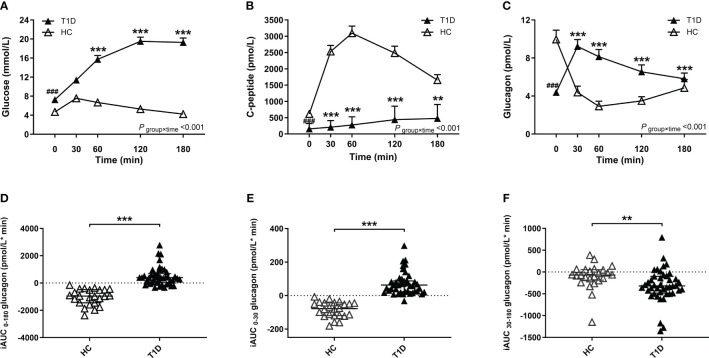
Results of the BMTT in the patients with T1D (n=43, filled triangles) and healthy control (n=24, open triangles). **(A–C)** The curve of plasma glucose, C-peptide and glucagon during the BMTT. * The repeated measured variables following the BMTT between two groups were investigated by generalized estimating equations with baseline measurement (0 min) as the covariates. * The simple effect of group was analyzed using the generalized estimation equation, ***P<0·001; **P<0·01. **^#^** The difference of baseline measurement was analyzed using the Mann-Whitney U test, **^###^***P*<0·001. **(D–F)** The incremental area under the curve of glucagon from 0 to 180 min, 0 to 30 min and 30 to 180 min during the BMTT, ***P<0·001; **P<0·01. BMTT, steamed bread meal tolerance test; HC, healthy control; T1D, type 1 diabetes; iAUC, incremental area under the curve.

Compared with healthy control group, patients with T1D showed higher glucose and lower C-peptide levels during the BMTT (**Figures 1A, B**) (**Supplementary Table 1**). We found that the fasting glucagon concentration in the healthy control was higher than T1D group (*P* < 0·001). A significant interaction between time and group was observed for glucagon after the BMTT (*P*_group×time_ <0·001), suggesting the different patterns of glucagon secretion after the BMTT between patients with T1D and healthy control group. After the BMTT, the healthy control group exhibited suppression of glucagon secretion, whereas patients with T1D showed elevated glucagon levels. The value of glucagon at each time point in T1D group were all higher than those in the healthy control group (**Figure 1C**) (**Supplementary Table 1**). The robustness of the glucagon level analyses between the T1D and HC groups was demonstrated in sensitivity analyses (**Supplementary Table 2**).

Meanwhile, the index of total glucagon response (iAUC_0-180_ glucagon) in the T1D group were higher than that in the healthy control group [398·00 (37·10; 689·00) vs. -960·00 (-1449·75; -493·25) pmol/L*min, *P* < 0·001]. Compared with the HC group, the early glucagon response (iAUC_0-30_ glucagon) was higher in the T1D group [62·80 (22·90; 87·10) vs. -78·05 (-119·50; -43·75) pmol/L*min, *P* < 0·001], while the late glucagon response (iAUC_30-180_ glucagon) was lower in the T1D group [-319·00 (-485·00; -100·00) vs. -69·00 (-185·50; 53·68) pmol/L*min, *P* = 0·002] (**Figures 1D–F**).

### 3.2 Different glucagon responses between T1D divided by stimulated C-peptide levels

To investigate the association between beta-cell function and glucagon response in patients with T1D, the participant was divided into the CPL and CPH group according to peak C-peptide level. The CPL group showed higher age and longer diabetes duration compared with the CPH group. No significant differences were observed in sex, age at diagnosis, BMI, HbA1c, and daily insulin dose between two groups (**Table 3**).

**Table 3:** Key clinical characteristics of patients with type 1 diabetes divided by peak C-peptide levels.

Characteristic	CPL group	CPH group	*P* value
Subjects, n	14	29	NA
Sex, male**/**female	6/8	18/11	0·235 ^a^
Age, years	49·67 (31·89; 63·18)	29·84 (21·42; 33·19)	0·003
Age at diagnosis, years	29·35 (20·34; 45·14)	25·48 (19·98; 31·85)	0·238
Duration of diabetes, years	7·00 (4·13; 21·25)	1·00 (0·25; 3·50)	<0·001
BMI, kg**/**m^2^	20·67 ± 2·51	20·17 ± 2·66	0·565
HbA1c, %	8·34 ± 1·63	9·73 ± 2·95	0·053
Daily insulin dose, U**/**day	31·84 ± 9·81	26·62 ± 7·09	0·053

Results were expressed as mean ± SD or median (25th; 75th). Variables were compared using the unpaired t test or Mann-Whitney U test; ^a^ Table was analyzed using Chi-squared test. NA, not applicable; CPL, C-peptide low; CPH, C-peptide high.

**Figure 2:**
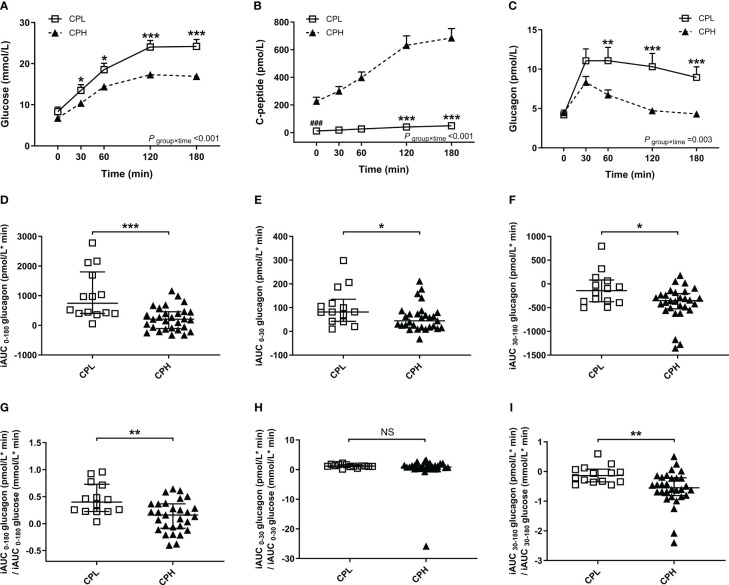
Results of the BMTT in the patients with T1D divide into subgroups according to peak C-peptide level: CPL group (peak C-peptide < 200pmol/L, n=14, open squares), CPH group (peak C-peptide ≥ 200pmol/L, n=29, filled triangles). **(A–C)** The curve of plasma glucose, C-peptide and glucagon during the BMTT. * The repeated measured variables following the BMTT between two groups were investigated by generalized estimating equations with baseline measurement (0 min) as the covariates. * The simple effect of group was analyzed using the generalized estimation equation; *P<0·05; **P<0·01; ***P<0·001. **^#^** The difference of baseline measurement was analyzed using the Mann-Whitney U test; **^###^***P*<0·001. **(D–F)** The incremental area under the curve of glucagon from 0 to 180min, 0 to 30 min and 30 to 180 min during the BMTT. **(G–I)** The ratio of iAUC glucagon to iAUC glucose from 0 to 180 min, 0 to 30 min and 30 to 180 min during the BMTT. *P<0·05; **P<0·01; ***P<0·001. BMTT, steamed bread meal tolerance test; T1D, type 1 diabetes; CPL, C-peptide low; CPH, C-peptide high; iAUC, incremental area under the curve; NS, nonsignificant.

Compared with the CPH group, the CPL group showed lower C-peptide and higher glucose levels during the BMTT (**Figures 2A, B**) (**Supplementary Table 3**). The fasting glucagon level was comparable between the CPL and CPH group (*P* = 0·708). There was a significant group by time interaction on glucagon during the BMTT between the CPL and CPH group (*P*_group×time_= 0·003), suggesting a difference in glucagon secretion after the BMTT between the CPL and CPH group. The value of glucagon at 60, 120 and 180 min in the CPL group were all higher than CPH group in response to the BMTT (**Figure 2C**) (**Supplementary Table 3**). Sensitivity analyses showed the robustness of the glucagon level analyses between the CPL and CPH groups (**Table 1**). In addition, peak glucagon was also higher in the CPL group compared with the CPH group [11·01 (7·80; 18·82) vs. 8·24 (5·68; 10·62) pmol/L, *P* = 0·018].

We also found that the glucagon response and glucagon response adjusted for glucose increment during the BMTT was different between the CPL and CPH group. Firstly, the index of total (iAUC_0-180_ glucagon), early (iAUC_0-30_ glucagon), and late (iAUC_30-180_ glucagon) glucagon response were all higher in the CPL group than the CPH group (**Figures 2D–F**). Secondly, the total and late glucagon response adjusted for glucose increment were both higher in the CPL group than in the CPH group (**Figures 2G, I**), while the early glucagon response adjusted for glucose increment was comparable between the two groups (**Figure 2H**) (**Supplementary Table 4**).

### 3.3 Relationship between peak C-peptide and glucagon secretion in response to the BMTT in individuals with T1D

Residual beta-cell function, defined as peak C-peptide concentration after the BMTT, was inversely related to the value of ▵glucagon at 120 and 180 min in patients with T1D (r = -0·476, *P* = 0·001; r = -0·530, *P* < 0·001, respectively). The peak C-peptide was inversely correlated with the iAUC _30-180_ glucagon (r= -0·450, *P* = 0·002), but not with the iAUC _0-30_ glucagon (r = -0·171, *P* = 0·274). Similarly, the peak C-peptide was also inversely correlated with the late glucagon response adjusted for glucose (iAUC _30-180_ glucagon/iAUC _30-180_ glucose) (r = -0·581, *P* < 0·001), but not with the early glucagon response adjusted for glucose (iAUC _0-30_ glucagon/iAUC _0-30_ glucose) (r= 0·044, *P =* 0·781).

Multiple linear regression analysis further showed that the peak C-peptide along with peak glucose affected the iAUC _30-180_ glucagon after adjusted for sex, age, BMI, duration of diabetes, and daily insulin dose (R^2^ = 0·335, *P* = 0·033). Moreover, the peak C-peptide, but not age and daily insulin dose, was also inversely related to the late glucagon response adjusted for glucose (iAUC _30-180_ glucagon/iAUC _30-180_ glucose) (R^2^ = 0·204, *P* = 0·029) (**Table 4**).

**Table 4:** Multiple linear regression coefficients for the association of the late glucagon response with peak C-peptide in type 1 diabetes after the BMTT.

	Dependent variables		Beta	Regression coefficient (95% CI) ^a^	*P* value
**Model 1**	iAUC 30-180 glucagon	Peak C-peptide	-0·47	-186·08 (-354·22 to -17·94)	0·031
		Sex	0·28	207·61 (-42·10 to 457·31)	0·100
		Age	-0·45	-176·07 (-358·45 to 6·31)	0·058
		BMI	0·08	33·13 (-121·14 to 187·40)	0·666
		Duration of diabetes	0·09	26·56 (-94·52 to 147·65)	0·659
		Daily insulin dose	-0·22	-119·07 (-308·35 to 70·21)	0·210
		Peak glucose	0·51	186·13 (34·75 to 337·50)	0·017
**Model 2**	iAUC 30-180 glucagon **/**iAUC 30-180 glucose	Peak C-peptide	-0·51	-0·29 (-0·50 to -0·08)	0·008
		Age	-0·08	-0·04 (-0·24 to 0·16)	0·672
		Daily insulin dose	-0·12	-0·09 (-0·34 to 0·16)	0·460

Multiple linear regression analysis was performed. In the model 1 where the dependent variable is iAUC 30-180 glucagon, F test = 2·515; R^2^ = 0·335; P = 0·033. In the model 2 where the dependent variable is iAUC 30-180 glucagon/iAUC 30-180 glucose, F test = 3·330; R^2^ = 0·204; P = 0·029. **^a^** Regression coefficient represent change in the iAUC 30-180 glucagon (pmol/L*min) or iAUC 30-180 glucagon/iAUC 30-180 glucose for per SD increase in the value of independent variables shown. BMTT, steamed bread meal tolerance test; Beta, standardized regression coefficient; CI, confidence interval; iAUC, incremental area under the curve; iAUC 30-180 glucagon/iAUC 30-180 glucose, the ratio of the glucagon iAUC from 30-180 min to the glucose iAUC from 30-180 min.

### 3.4 Relationship between glucagon secretion and glucose excursion in response to the BMTT in individuals with T1D

In order to determine the effect of glucagon response on the glucose increment in patients with T1D, we calculated the association between the paired ▵Glucagon and ▵Glucose during the BMTT. We found that the value of ▵Glucagon at 30, 60, 120, and 180 min were all positively correlated with the value of ▵Glucose at the same time point (**Supplementary Figure 2**).

We also investigated the effect of glucagon response on glucose increment in patients with T1D after the BMTT. The index of late glucagon response (iAUC _30-180_ glucagon) were positive related to the value of ▵Glucose 180 min (r = 0·370, *P* = 0·014). From the multiple linear regression analysis, we found that the peak C-peptide along with iAUC _30-180_ glucagon affected the value of ▵Glucose 180 min after adjusted for sex, age at diagnosis, and daily insulin dose (R^2^ = 0·720, *P* < 0·001) (**Table 5**).

**Table 5:** Multiple linear regression coefficients for the association of the glucagon response with ▵glucose 180 in type 1 diabetes after the BMTT.

	Beta	Regression coefficient (95% CI) ^a^	*P* value
Peak C-peptide	-0·27	-1·29 (-2·40 to -0·18)	0·024
iAUC 0-30 glucagon	0·46	2·01 (1·01 to 3·02)	<0·001
iAUC 30-180 glucagon	0·39	1·73 (0·71 to 2·76)	0·002
Sex	-0·16	-1·44 (-3·23 to 0·35)	0·111
Age at diagnosis	0·27	1·30 (0·28 to 2·32)	0·014
Daily insulin dose	-0·07	-0·48 (-1·81 to 0·86)	0·472

Multiple regression analysis was performed, F test = 15·442; R^2^ = 0·720; P < 0·001; **^a^** Regression coefficient represent change in the ▵glucose (mmol/L) for per SD increase in the value of independent variables shown. BMTT, steamed bread meal tolerance test; Beta, standardized regression coefficient; CI, confidence interval; iAUC, incremental area under the curve.

## 4 Discussion

Islet beta cells in patient with T1D exhibit ethnic heterogeneity, with better beta cell function observed in Chinese patients with T1D. The functional changes in alpha cells in patients with T1D with different stimulated C-peptide levels remain poorly elucidated. Our research aimed to investigate the association between insulin and the phase of glucagon secretion in patients with T1D exhibiting different stimulated C-peptide levels. Our findings can be summarized as follows: 1) stimulated C-peptide levels affected the paradoxical increase in postprandial glucagon secretion in patients with T1D, especially the elevated extent of the late glucagon response; 2) the late glucagon response affects the glucose increment in 180 min.

Glucose homeostasis is primarily regulated primarily by the two key regulatory hormones insulin and glucagon. Many studies have shown that the metabolic expression of uncontrolled diabetes is the consequence of abnormalities in these two hormones (22). During fasting, the balance between insulin and glucagon is crucial in preventing hypoglycemia. In individuals without diabetes, the basal glucagon concentration maintains approximately half of the basal hepatic glucose production, which regulates fasting plasma glucose levels (23). Brown et al. reported normal fasting glucagon concentrations for up to 12 months following the diagnosis of T1D (9). However, other studies have indicated slightly lower fasting glucagon levels in patients with T1D compared to healthy subjects (10, 20). In contrast, our findings revealed a significantly reduction in fasting glucagon concentration among patients with T1D compared to healthy controls. This insufficient fasting glucagon levels partly supported the susceptibility to hypoglycemia in patients with T1D. The persistence of C-peptide secretion exhibits considerable variability widely in individuals diagnosed as T1D (24, 25). Several studies have confirmed inadequate glucagon secretion during hypoglycemia in patients with T1D, with elevated glucagon correlating with beta cell function (26, 27). Our study did not identify in fasting glucagon levels in patients with T1D exhibiting different C-peptide levels, probably due to the fact that fasting is not accurately reflect the hypoglycemic status.

In addition to the abnormal fasting glucagon levels, the increasing postprandial glucagon in T1D after oral glucose and mixed-meal intake has been demonstrated in several studies (9, 28). Our results support these previous reports by revealing elevated glucagon levels at 30-180min and total glucagon response during the BMTT in patients with T1D. Local insulin secretion in intra-islets plays a critical role in suppressing glucagon secretion during hyperglycemia (29). The Diabetes Control and Complications Trial (DCCT) demonstrated that a stimulated C-peptide value ≥ 200 pmol/L showed benefits for glycemic control and a minimal number of complications (30). Therefore, we set the threshold of 200 pmol/L for C-peptide to compare the differences in glucagon secretion in patients with T1D exhibiting different residual beta cell function. Our results showed that stimulated C-peptide levels in T1D did affect the paradoxical increase in glucagon secretion after the BMTT. Patients with peak C-peptide below 200 pmol/L exhibited higher peak glucagon and glucagon at 60-180 min. Furthermore, the value of ▵glucagon at 120 and 180 min was negatively correlated with peak C-peptide in response to the BMTT.

Our findings above are consistent with previous research indicating a decrease C-peptide levels and an increase in postprandial glucagon levels as T1D progresses (9, 11, 31). However, our results contrast with the previous studies that T1D individuals with different residual C-peptide had comparable glucagon response after oral glucose challenge (12, 13, 32). The observed discrepancy may be attributed to the difference in stimulation components. The previous study used a mixed meal tolerance test (MMTT) consisted of 50-72% carbohydrate, 18-37% protein and 10-14% fat, while our participant underwent the BMTT, which consisted of a high amount of 90·3% carbohydrate and a low proportion of 8·4% protein and 1·2% fat. It has been reported that alpha cells exhibit varying degrees of sensitivity to different stimuli. In healthy individuals, glucose administration inhibits glucagon secretion, while protein intake activates glucagon secretion (33). The inclusion of protein in the diet of patients with T1D has a significant impact on the total concentration and peak levels of glucagon (34). In fact, several amino acids, such as alanine, arginine, cystine, and proline, have been reported to stimulate glucagon secretion in rodents. Although the glucagon-stimulating potency of individual amino acids is not yet known in humans (29), it is plausible that amino acids present in the diet may stimulate glucagon secretion.

Glucose-stimulated insulin secretion is characterized by a transient first phase followed by a sustained second phase (35). Given the inter-regulatory role of islet beta and alpha cells (29), it is reasonable to hypothesize that glucose-stimulated glucagon secretion may be a biphasic pattern. Two studies have utilized a 30-min time points after glucose intake to differentiate between early and late glucagon response in diabetic patients (14, 15), and highlighted the importance of early glucagon response in T2D, and had suggested that individuals with newly diagnosed T2D exhibited impairment only in early glucose-stimulated glucagon suppression (15). Similarly, our study revealed the iAUC of glucagon from 0-30 was higher in T1D than healthy control, suggesting that T1D also has an impairment in early suppression of glucagon stimulated by glucose compared with subjects with normal glucose tolerance. Our study found that the iAUC of glucagon from 30-180 min was higher in patients with peak C-peptide below 200 pmol/L, and this increase was negatively correlated with peak C-peptide. In contrast, there was no linear correlation between the iAUC of glucagon from 0-30 min and peak C-peptide levels, although the iAUC of glucagon from 0-30 min was also higher in patients with peak C-peptide below 200 pmol/L. Therefore, we identified the importance of the late glucagon response in T1D, and proposed, for the first time, that C-peptide levels influence the late glucagon response in T1D individuals following the BMTT in our present study.

Glucose has a direct effect on glucagon secretion, with glucose administration inhibiting glucagon secretion in healthy individuals (33). However, the effect of glucose on glucagon secretion and the underlying mechanisms are complex and disputed. Salehi et al. found that elevated blood glucose levels elicited a dose-dependent stimulation of glucagon release (36). Vieira et al. found an inhibition of glucagon secretion from isolated mouse islets within the glucose range of 4 to 20 mmol/L, while glucagon secretion increased when glucose levels exceeded 20 mmol/L (37). Wang et al. reported an increase in glucagon secretion from the pancreases of insulin deficient T1D rats when perfused glucose concentration was raised from 5 to 25 mmol/L (38). Glucose elevations that are not accompanied by a parallel increase in insulin levels will result in hyperglycemia, which in turn stimulates glucagon secretion in a counter-regulatory manner. In our present study, we found higher glucose trend following the BMTT in patients with peak C-peptide below 200 pmol/L, and the mean value of glucose at 120-180 min were above 20 mmol/L. In addition, the peak C-peptide along with peak glucose were correlate with the iAUC _30-180_ glucagon in the multiple linear regression. These finding suggest that excessive postprandial glucose levels following the BMTT in the patients with peak C-peptide below 200 pmol/L may further promote postprandial glucagon secretion. However, the interaction between glucose and glucagon is complex, and as our study is a cross-sectional study, it is difficult to establish a causal association. Further investigations are warranted to validate these findings.

One remaining question is whether there is a differential glucagon response adjusted for glucose increment after oral glucose intake, and whether this response is dependent on the stimulated C-peptide levels in individuals with T1D. Kramer et al. found that glycemic normalization prior to oral glucose ingestion did not change the suppression of glucagon per glucose increment in long-duration T1D during an oral glucose tolerance test (21). In our present study, we utilized the ratio of iAUC glucagon to iAUC glucose to estimate the time course of glucose-induced glucagon secretion adjusted for glucose increments in patients with T1D exhibiting different stimulated C-peptide levels. The glucagon level adjusted for glucose increments was almost identical to those prior to correction. We found that the late glucagon response adjusted for glucose increment were higher in patients with T1D who had lower peak C-peptide levels, and these responses was negatively correlated with peak C-peptide levels. These results further highlighted the significant of the late glucagon response in individuals with T1D with different stimulated C-peptide levels.

In our present study, we found that glucose at 30-180 min following the BMTT was higher in patients with peak C-peptide < 200 pmol/L. Additionally, the pair of parameter between ▵glucose and ▵glucagon at 30-180 min was positively correlated. Importantly, the late glucagon response and peak C-peptide levels were correlated with the postprandial glucose increment after adjusting for confounding factors. These results suggested that patients with T1D exhibiting low stimulated C-peptide levels had greater paradoxical increase in postprandial glucagon secretion, especially in the late glucagon response. The lack of adequate insulin secretion along with excessive glucagon secretion usually lead to hyperglycemic state.

To our knowledge, this study is one of the few investigations examining the effect of stimulated C-peptide levels on glucagon secretion in Chinse patients with T1D. Moreover, it is the first study to report a significant influence of C-peptide levels on the glucagon response during the late postprandial phase in individuals with T1D. Notably, exogenously insulin, unlike endogenously insulin, is insufficient to provide high concentrations of insulin within the islets of Langerhans, resulting to elevated glucagon levels and further complicating glycemic control in T1D (39). Recent years have witnessed the development of various classes of glucose-lowering medications. Targeting excessive postprandial glucagon secretion represents a potential strategy to mitigate hyperglycemia in individuals with T1D. A phase I trial showed that a single dose of a glucagon receptor antibody (volagidemab) decreased insulin requirements and improved glycemic control in patients with T1D (40). Furthermore, in a subsequent phase II clinical trial, 12-week adjunctive therapy with volagidemab was associated with decreased HbA1c and stable insulin dose (41). Therefore, this study has important clinical and public health implications as it provides insight into the role of postprandial hyperglycemia in T1D. Furthermore, it may provide a theoretical basis for the development of glucagon-based therapeutic approaches for T1D.

Our study has some limitations which include a small sample size and an open-label, cross-sectional design. Secondly, glucagon-like peptide 1 (GLP-1) could inhibit glucagon release in a glucose-dependent manner. The absence of evaluation of GLP-1 in our study hinders our understanding of the precise role of stimulated C-peptide levels in glucagon secretion under the influence of other potential factors in patients with T1D. Thirdly, several previous studies found that sex influences postprandial glucagon secretion in healthy individuals (42) and patients with T1D (11). However, our study did not find a significant impact of sex on the conclusion that stimulated C-peptide affects postprandial glucagon secretion in patients with T1D. Further research is required to confirm this conclusion and explore the underlying mechanisms. Recent evidence has demonstrated a ‘pancreatic’ 29-amino-acid glucagon in patients who had undergone totally pancreatectomy, indicating the existence of extrapancreatic glucagon (43); Unfortunately, due to the inclusion of participants without any history of pancreatic and intestinal surgeries, we were unable to distinguish whether the glucagon detected in our study contained exocrine pancreatic secretion. The physiology of exocrine glucagon in T1D remains unclear.

## 5 Conclusion

In conclusion, this study investigated the regulation of glucagon secretion in individuals with T1D and demonstrated that stimulated C-peptide levels play a role in the paradoxical increase of postprandial glucagon secretion, particularly in the late glucagon response. This exaggerated postprandial glucagon secretion contributes to the elevation of blood glucose after oral glucose intake. Our findings have significant implications for understanding the pathophysiology of postprandial hyperglycemia in T1D and may provide the development of glucagon-based therapies for this condition.

## Data availability statement

The original contributions presented in the study are included in the article/**Supplementary Material**. Further inquiries can be directed to the corresponding author.

## Ethics statement

The studies involving humans were approved by The local ethics committee of the First Affiliated Hospital of Nanjing Medical University. The studies were conducted in accordance with the local legislation and institutional requirements. The participants provided their written informed consent to participate in this study.

## Author contributions

LZ: Data curation, Formal analysis, Methodology, Writing – original draft. YQ: Data curation, Methodology, Project administration, Writing – review & editing. YH: Data curation, Writing – review & editing. QH: Data curation, Writing – review & editing. QW: Data curation, Writing – review & editing. XW: Data curation, Writing – review & editing. MZ: Funding acquisition, Resources, Supervision, Validation, Writing – review & editing.
