# Supplementary material for: Abnormal late postprandial glucagon response in type 1 diabetes is a function of differences in stimulated C-peptide concentrations
Source: Front Endocrinol (Lausanne). 2024 Aug 1;15:1419329. doi: 10.3389/fendo.2024.1419329 (PMC11324558; doi:10.3389/fendo.2024.1419329)
Supplement: Supplementary file 1 [file DataSheet_1.docx]

**Supplemental Material**

| **Content** | **Page** |
| --- | --- |
| **Supplementary Figure 1.** Flow diagram of participants through the study. | 1 |
| **Supplementary Figure 2.** Relationship between pairs of △glucose and △glucagon at 30 min, 60 min, 120 min, 180 min during the BMTT in patients with type 1 diabetes. | 2 |
| **Supplementary Table 1.** Results of the steamed bread meal tolerance test in participants with type 1 diabetes and healthy control. | 3 |
| **Supplementary Table 2.** Sensitivity analyses of the glucagon levels during the BMTT in participants with type 1 diabetes and healthy control. | 4 |
| **Supplementary Table 3.** Result of the steamed bread meal tolerance test in participants with type 1 diabetes divided by peak C-peptide levels. | 5 |
| **Supplementary Table 4.** Result of the glucagon response in participants with type 1 diabetes divided by peak C-peptide levels. | 6 |

**
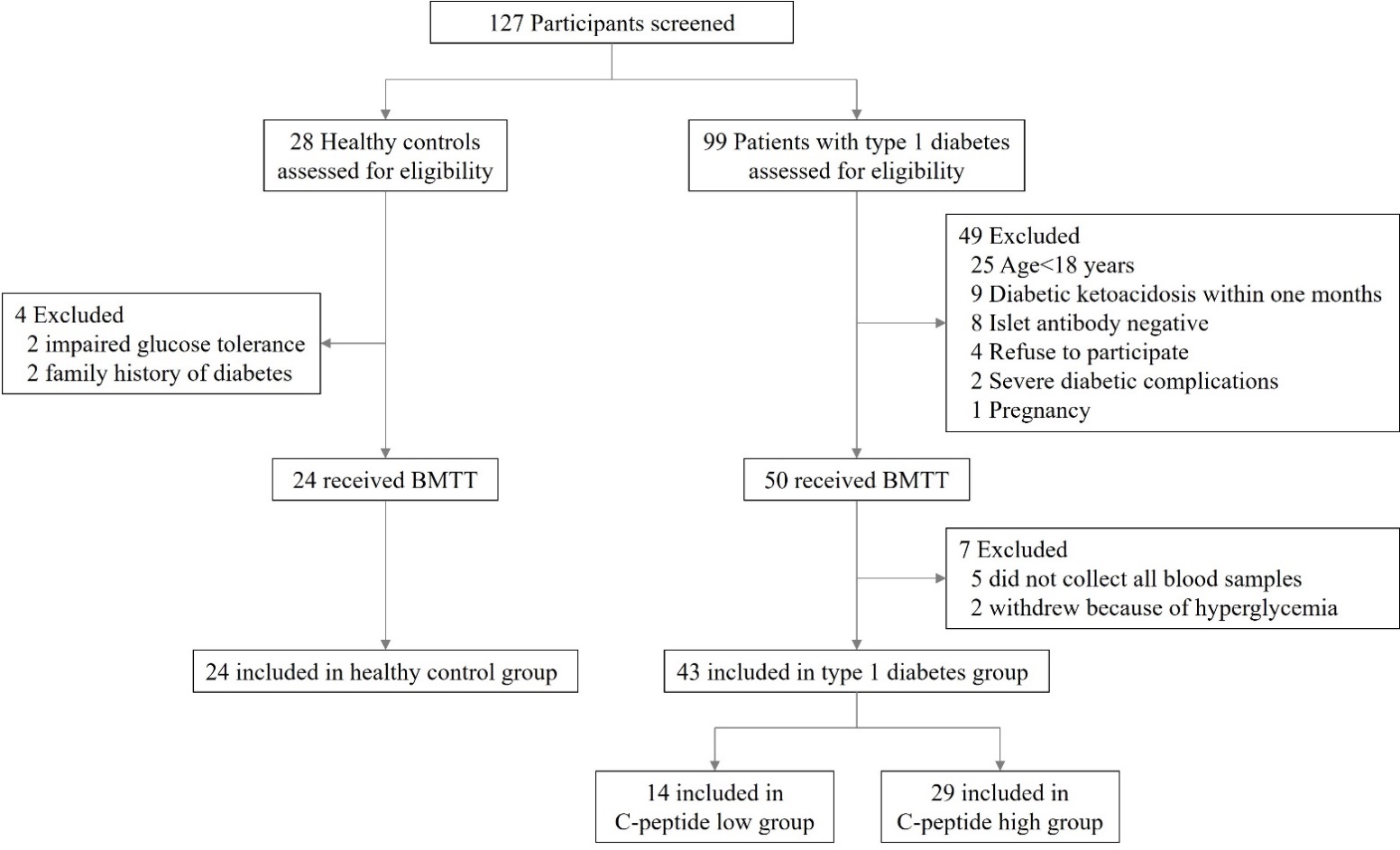
**

**Supplementary Figure 1.** **Flow diagram of participants through the study**. C-peptide group was determined according to the peak serum C-peptide level after BMTT. BMTT, steamed bread meal tolerance test.


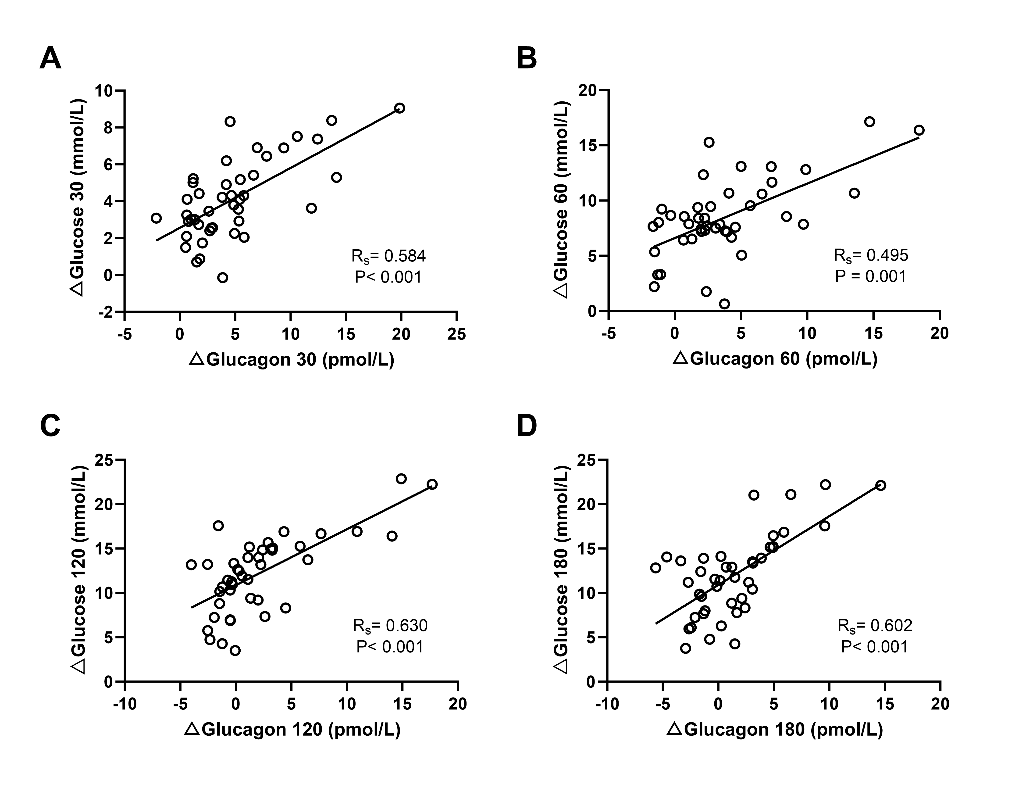


**Supplementary Figure 2.** Relationship between pairs of △glucose and △glucagon at (A) 30 min, (B) 60 min, (C) 120 min, (D) 180 min during the BMTT in patients with type 1 diabetes (n=43). Abbreviations: BMTT, steamed bread meal tolerance test.

**Supplementary Table 1.** Results of the steamed bread meal tolerance test in participants with type 1 diabetes and healthy control.

|  | **T1D group**  **(n=43)** | **HC group**  **(n=24)** | **Group×time interaction effect** | **T1D group vs. HC group** | |
| --- | --- | --- | --- | --- | --- |
|  |  |  |  | **Adjusted mean difference (95% CI)** | ***P* value** |
| **Glucose [median (25th; 75th)], mmol/L** | | | | | |
| 0 min | 6·96 (4·92; 9·15) | 4·71 (4·43; 4·86) | <0·001 |  |  |
| 30 min | 10·30 (7·93; 13·22) | 7·50 (6·54; 8·50) |  | 0·60 (-0·33 to 1·53) | 0·207 |
| 60 min | 14·55 (11·44; 19·74) | 6·59 (5·16; 7·77) |  | 5·82 (4·55 to 7·09) | <0·001 |
| 120 min | 18·71 (15·61; 22·47) | 5·17 (4·45; 6·07) |  | 10·97 (9·64 to 12·31) | <0·001 |
| 180 min | 17·68 (15·24; 21·99) | 3·90 (3·11; 5·10) |  | 11·82 (10·41 to 13·22) | <0·001 |
| **C-peptide [median (25th; 75th)], pmol/L** | | | | | |
| 0 min | 95·20 (9·99; 252·00) | 543·28 (396·84; 720·76) | <0·001 |  |  |
| 30 min | 155·80 (26·89; 295·20) | 2597·04 (1753·87; 3057·50) |  | -1626·46 (-2015·22 to -1237·70) | <0·001 |
| 60 min | 227·60 (51·95; 438·00) | 3289·67 (2083·12; 3943·77) |  | -2119·67 (-2600·00 to -1639·34) | <0·001 |
| 120 min | 372·00 (87·79; 710·20) | 2298·11 (1767·75; 2813·89) |  | -1350·63 (-1714·29 to -986·96) | <0·001 |
| 180 min | 429·80 (98·74; 759·92) | 1524·85 (817·90; 2220·36) |  | -479·71 (-787·83 to -171·59) | 0·002 |
| **Glucagon [median (25th; 75th)], pmol/L** | | | | | |
| 0 min | 4·24 (3·14; 5·67) | 9·63 (6·15; 12·24) | <0·001 |  |  |
| 30 min | 8·39 (5·72; 11·63) | 3·48 (2·39; 5·41) |  | 7·44 (5·85 to 9·02) | <0·001 |
| 60 min | 7·01 (4·70; 10·78) | 2·02 (1·33; 3·56) |  | 7·81 (6·19 to 9·43) | <0·001 |
| 120 min | 4·73 (3·46; 7·69) | 3·11 (1·72; 4·32) |  | 5·65 (3·71 to 7·60) | <0·001 |
| 180 min | 5·01 (3·07; 7·34) | 3·23 (2·06; 7·09) |  | 3·55 (1·72 to 5·39) | <0·001 |

The repeated measured variables following the BMTT between two groups were investigated by generalized estimating equations with baseline measurement (0 min) as the covariates. A significant group×time interaction indicated a significant difference for the given variables between groups during the BMTT. T1D, type 1 diabetes; HC, healthy control.

**Supplementary Table 2.** Sensitivity analyses of the glucagon levels during the BMTT in participants with type 1 diabetes and healthy control.

|  | **Glucagon [median (25th; 75th)], pmol/L** | | **Group×time interaction effect** | **T1D group vs. HC group** | |
| --- | --- | --- | --- | --- | --- |
|  | **T1D group**  **(n=43)** | **HC group**  **(n=24)** |  | **Adjusted mean difference (95% CI)** | ***P* value** |
| **Multiple imputation** | | | | | |
| 0 min | 4·24 (3·14; 5·67) | 9·63 (6·15; 12·24) | <0·001 |  |  |
| 30 min | 8·39 (5·72; 11·63) | 3·48 (2·39; 5·41) |  | 7·44 (5·85 to 9·02) | <0·001 |
| 60 min | 7·01 (4·70; 10·78) | 2·02 (1·33; 3·56) |  | 7·81 (6·19 to 9·43) | <0·001 |
| 120 min | 4·73 (3·46; 7·69) | 3·11 (1·72; 4·32) |  | 5·65 (3·71 to 7·60) | <0·001 |
| 180 min | 5·01 (3·07; 7·34) | 3·23 (2·06; 7·09) |  | 3·55 (1·72 to 5·39) | <0·001 |
| **Multiple imputation with adjustment for age and baseline measurement** | | | | | |
| 0 min | 4·24 (3·14; 5·67) | 9·63 (6·15; 12·24) | <0·001 |  |  |
| 30 min | 8·39 (5·72; 11·63) | 3·48 (2·39; 5·41) |  | 5·96 (4·29 to 7·63) | <0·001 |
| 60 min | 7·01 (4·70; 10·78) | 2·02 (1·33; 3·56) |  | 6·34 (4·83 to 7·84) | <0·001 |
| 120 min | 4·73 (3·46; 7·69) | 3·11 (1·72; 4·32) |  | 4·18 (2·47 to 5·88) | <0·001 |
| 180 min | 5·01 (3·07; 7·34) | 3·23 (2·06; 7·09) |  | 2·08 (0·31 to 3·85) | 0·021 |

In the sensitivity analyses, the robustness of the results was assessed using 2 different analytical approaches. The repeated measured glucagon levels following the BMTT between two groups were investigated by generalized estimating equations. The first approach included baseline measurement (0 min) as covariates; The second approach included age and baseline measurement (0 min) as covariates. A significant group×time interaction indicated a significant difference for glucagon levels between two groups during the BMTT in all 2 approaches. CI, confidence interval; T1D, type 1 diabetes; HC, healthy control.

**Supplementary Table 3.** Result of the steamed bread meal tolerance test in participants with type 1 diabetes divided by peak C-peptide levels.

|  | **CPL group**  **(n=14)** | **CPH group**  **(n=29)** | **Group×time interaction effect** | **CPL group vs. CPH group** | |
| --- | --- | --- | --- | --- | --- |
|  |  |  |  | **Adjusted mean difference (95% CI)** | ***P* value** |
| **Glucose [median (25th; 75th)], mmol/L** | | | | | |
| 0 min | 7·85 (4·87; 10·79) | 6·96 (4·92; 8·12) | <0·001 |  |  |
| 30 min | 13·01 (8·60; 19·35) | 10·30 (7·76; 12·55) |  | 1·32 (0·09 to 2·54) | 0·035 |
| 60 min | 19·12 (12·70; 23·39) | 14·34 (10·83; 16·06) |  | 2·38 (0·31 to 4·46) | 0·024 |
| 120 min | 23·24 (17·97; 29·66) | 16·44 (14·10; 19·69) |  | 4·97 (2·89 to 7·05) | <0·001 |
| 180 min | 22·87 (18·36; 29·76) | 16·94 (14·30; 18·86) |  | 5·51 (3·32 to 7·71) | <0·001 |
| **C-peptide [median (25th; 75th)], pmol/L** | | | | | |
| 0 min | 3·33 (3·33; 12·22) ^a^ | 182·80 (94·45; 331·50) | <0·001 |  |  |
| 30 min | 3·33 (3·33; 31·06) ^a^ | 245·00 (145·50; 459·95) |  | 36·64 (-36·08 to 109·35) | 0·323 |
| 60 min | 3·33 (3·33; 55·75) ^a^ | 384·60 (213·65; 572·15) |  | -52·98 (-135·52 to 29·57) | 0·208 |
| 120 min | 3·33 (3·33; 96·32) ^a^ | 521·10 (358·80; 818·96) |  | -272·90 (-376·16 to -167·63) | <0·001 |
| 180 min | 3·33 (3·33; 107·58) ^a^ | 610·60 (395·45; 859·05) |  | -317·64 (-424·43 to -210·85) | <0·001 |
| **Glucagon (mean±SD), pmol/L** | | | | | |
| 0 min | 4·20±2·24 | 4·47±2·28 | 0·003 |  |  |
| 30 min | 11·05±5·66 | 8·32±4·03 |  | 2·93 (-0·01 to 5·87) | 0·051 |
| 60 min | 11·07±6·31 | 6·72±3·41 |  | 4·55 (1·53 to 7·57) | 0·003 |
| 120 min | 10·31±6·31 | 4·72±2·37 |  | 5·79 (2·68 to 8·90) | <0·001 |
| 180 min | 8·95±5·03 | 4·31±2·09 |  | 4·85 (2·41 to 7·29) | <0·001 |

The repeated measured variables following the BMTT between two groups were investigated by generalized estimating equations with baseline measurement (0 min) as the covariates. A significant group×time interaction indicated a significant difference for the given variables between groups during the BMTT. ^a^ The value of 3·33 represent the value of C-peptide below detectable limit. CPL, C-peptide low; CPH, C-peptide high.

**Supplementary Table 4.** Result of the glucagon response in participants with type 1 diabetes divided by peak C-peptide levels.

|  | **CPL group** | **CPH group** | ***P* value** |
| --- | --- | --- | --- |
| **Glucagon response** |  |  |  |
| iAUC 0-180 glucagon, pmol/L* min | 746·00 (400·25; 1800·00) | 208·00 (-107·75; 459·50) | 0·001 |
| iAUC 0-30 glucagon, pmol/L* min | 81·65 (42·28; 135·25) | 44·40 (18·05; 76·75) | 0·026 |
| iAUC 30-180 glucagon, pmol/L* min | -142·00 (-376·00; 81·45) | -353·00 (-526·00; -206·00) | 0·036 |
| **Glucagon response adjusted for glucose** |  |  |  |
| iAUC 0-180 glucagon / iAUC 0-180 glucose | 0·45±0·29 | 0·14±0·29 | 0·002 |
| iAUC 0-30 glucagon / iAUC 0-30 glucose | 1·18 (1·05; 1·65) | 0·86 (0·32; 1·45) | 0·325 |
| iAUC 30-180 glucagon / iAUC 30-180 glucose | -0·14 (-0·35; 0·08) | -0·60 (-0·82; -0·22) | 0·001 |

Results were expressed as mean ± SD or median (25th; 75th). *P* value was analyzed using the unpaired t-test or Mann-Whitney U test. CPL, C-peptide low; CPH, C-peptide high; iAUC, incremental areas under the curve.
